# Antimicrobial properties of tomato leaves, stems, and fruit and their relationship to chemical composition

**DOI:** 10.1186/s12906-021-03391-2

**Published:** 2021-09-13

**Authors:** Christina C. Tam, Kevin Nguyen, Daniel Nguyen, Sabrina Hamada, Okhun Kwon, Irene Kuang, Steven Gong, Sydney Escobar, Max Liu, Jihwan Kim, Tiffany Hou, Justin Tam, Luisa W. Cheng, Jong H. Kim, Kirkwood M. Land, Mendel Friedman

**Affiliations:** 1 Foodborne Toxin Detection and Prevention Research Unit, Agricultural Research Service, United States Department of Agriculture, Albany, California 94710 USA; 2 Department of Biological Sciences, University of the Pacific, Stockton, California 95211 USA; 3 Healthy Processed Foods Research Unit, Agricultural Research Service, United States Department of Agriculture, Albany, California 94710 USA

**Keywords:** *Trichomonas vaginalis*, *Tritrichomonas foetus*, *Lactobacilli*, bacteria, fungi, Infection, Trichomoniasis, Inhibition, Leaves, Stems, Tomato, Tomatine, Phenolic, Flavonoid

## Abstract

**Background:**

We previously reported that the tomato glycoalkaloid tomatine inhibited the growth of *Trichomonas vaginalis* strain G3, *Tritrichomonas foetus* strain D1, and *Tritrichomonas foetus*-like strain C1 that cause disease in humans and farm and domesticated animals. The increasing prevalence of antibiotic resistance requires development of new tools to enhance or replace medicinal antibiotics.

**Methods:**

Wild tomato plants were harvested and divided into leaves, stems, and fruit of different colors: green, yellow, and red. Samples were freeze dried and ground with a handheld mill. The resulting powders were evaluated for their potential anti-microbial effects on protozoan parasites, bacteria, and fungi. A concentration of 0.02% (w/v) was used for the inhibition of protozoan parasites. A high concentration of 10% (w/v) solution was tested for bacteria and fungi as an initial screen to evaluate potential anti-microbial activity and results using this high concentration limits its clinical relevance.

**Results:**

Natural powders derived from various parts of tomato plants were all effective in inhibiting the growth of the three trichomonads to varying degrees. Test samples from leaves, stems, and immature ‘green’ tomato peels and fruit, all containing tomatine, were more effective as an inhibitor of the D1 strain than those prepared from yellow and red tomato peels which lack tomatine. Chlorogenic acid and quercetin glycosides were present in all parts of the plant and fruit, while caffeic acid was only found in the fruit peels. Any correlation between plant components and inhibition of the G3 and C1 strains was not apparent, although all the powders were variably effective. Tomato leaf was the most effective powder in all strains, and was also the highest in tomatine. *S. enterica* showed a minor susceptibility while *B. cereus* and *C. albicans* fungi both showed a significant growth inhibition with some of the test powders. The powders inhibited growth of the pathogens without affecting beneficial lactobacilli found in the normal flora of the vagina.

**Conclusions:**

The results suggest that powders prepared from tomato leaves, stems, and green tomato peels and to a lesser extent from peels from yellow and red tomatoes offer potential multiple health benefits against infections caused by pathogenic protozoa, bacteria, and fungi, without affecting beneficial lactobacilli that also reside in the normal flora of the vagina.

## Background

Infection by the parasitic protozoan *Trichomonas vaginalis* in humans causes the sexually transmitted disease (STD) trichomoniasis, reported to be the most common non-viral transmitted infection in the world [1, 2]. Strains of *Tritrichomonas foetus* are reported to cause the disease of trichomonosis in farm animals (cattle, bulls, and pigs) [3–6], as well as in domestic animals (cats and dogs) [6, 7]. In cows, the disease causes failed pregnancies and infected cows are usually culled. In domesticated cats, the disease infects the gastrointestinal tract, causing diarrhea, and is transmitted by the oral-fecal route.

Because of increasing rates of clinical resistance to the widely used drug metronidazole, new treatments are needed to replace or to complement current available therapies. The need for new treatments is illustrated by a publication from the National Institute of Allergy and Infectious Diseases, National Institute of Health (NIH) that emphasizes the need for new therapeutics to help overcome the global epidemic of sexually transmitted infections, including trichomoniasis [1].

As part of an effort to discover the efficacy of safe food extracts and their bioactive constituents against pathogenic trichomonads, we previously reported on the anti-trichomonad effects of potato and tomato glycoalkaloids, potato peels, and of black tea and other food-compatible compounds and extracts against three trichomonad parasites [8–12], reviewed in [12].

To help meet the need to develop new effective therapeutic agents, the objective of the present study is to evaluate anti-trichomonad, antibacterial, and anti-fungal properties of powders prepared from leaves, stems, and tomatoes harvested from a growing plant, and correlate this inhibitory activity to their composition of a separate set of test powders, as determined previously using high-performance-liquid chromatography/mass spectrometry (HPLC/MS). The results of the described efforts suggest that several tomato-plant-derived powders could potentially replace or enhance the therapeutic potency of metronidazole.

## Methods

### Materials

#### Source of tomato plant

The wild, drought-resistant tomato plant was obtained from a residential garden in California with the permission of the owners. The plant and plant parts used in this study were harvested in the summer of 2018. This plant bears small fruit at different stages of maturity approximately the size of cherry tomatoes ranging from green (immature), yellow, then reddish colors. Informal determination via morphological identification by Prof. Dina St. Clair at the University of California, Davis indicates that “It looks like a small fruited version of cultivated tomato (*S. lycopersicum*) although it’s not likely to be a cultivar or variety per se. It may be a “weedy” or naturalized form. They are common in fields where tomatoes have been grown. Genomic analysis and typing of this plant have not been undertaken therefore this identification is preliminary. We plan a genetic analysis using newly grown plants. Materials will be stored in our laboratory, United States Department of Agriculture, Foodborne Toxin Detection and Prevention Research Unit at Albany, CA, 94710, USA.

#### Sources of trichomonad parasites

*Trichomonas vaginalis* strain G3 was obtained from Patricia Johnson, University of California at Los Angeles, CA, USA. *Tritrichomonas foetus* strain D1 was obtained from Lynette Corbeil, University of California at San Diego, School of Medicine, La Jolla, CA, USA, and feline *Tritrichomonas foetus*-like organism (strain C1) from Stanley Marks, University of California at Davis, School of Veterinary Medicine, Davis, CA, USA. All of these protozoa were cultured according to methods previously described [9]. The Institutional Biosafety Committee at the University of Pacific approved the use of these pathogenic organisms.

#### Sources of pathogenic and nonpathogenic Bacteria and Fungi

The pathogenic and non-pathogenic bacteria and pathogenic fungi were obtained from the in-house United States Department of Agriculture (USDA) collection or from the American Type Culture Collection (ATCC, Manassas, VA, USA). The Institutional Biosafety Committee at the USDA approved the use of these non-pathogenic and pathogenic organisms.

#### Preparation of powders from the tomato plant

To demonstrate proof-of-principle, a tomato plant of unknown origin was randomly selected for this study from a garden of a private residence in Solano County, CA. Tomato leaves, stems, and green, yellow, and red tomatoes were harvested from the tomato plant as described previously [13]. Briefly, the harvested samples were randomly divided into several samples. The three types of tomato fruit were peeled by placing them in boiling water for 1 min followed by ice water for another minute, then drying with absorbent paper tissue. The tomatoes were then peeled using a knife. The leaves, stems, and peels were freeze-dried. The dried samples were ground to fine powders using an electric coffee grinder (Krups, Millville, New Jersey, USA). The powders were used to determine their inhibitory activities against: (a) three parasitic trichomonad strains (human *Trichomonas vaginalis* G3, feline *Tritrichomonas foetus*-like C1, and bovine *Tritrichomonas foetus* D1); (b) four pathogenic bacteria (*S. enterica*, *L. monocytogenes*, *S. aureus*, and *B. cereus*); (c) four nonpathogenic bacteria (*E. coli* K12 used as replacement for pathogenic *E. coli*, *L. acidophilus*, *L. rhamnosus* GG, and *L. reuteri*); and (d) two pathogenic fungi (*Aspergillus fumigatus* and *C. albicans*), as described below.

#### Analytical aspects

The composition of the powders was determined by high-performance liquid chromatography (HPLC) and fast atom bombardment mass spetrometry (FAB-MS) using using a Hitachi 7000 liquid chromatography system with UV/vis detector and a JMS-700 double focusing mass spectrometer, as described in detail elsewhere [13]. Each peak was identified by comparing the absorption spectra, retention times, and chromatographic peak areas of unknown compounds in the analyzed samples to those of standards analyzed under the same conditions. Statistical significance of the difference between samples was tested using the Tukey test, with *p* ≤ 0.5.

### Trichomonad growth inhibition assay

#### Protozoan parasite inhibition assays

Stock solutions of the plant powders (10% w/v) were prepared by solubilizing in a solution of 1:1 autoclaved water to DMSO (density of DMSO approximated to 1 g/mL) as follows. The powders were first dissolved or suspended in DMSO. The water was then added, and the powder was resuspended and used in bioassays immediately at 0.02% w/v. Cultures of the G3 strain of *T. vaginalis* and C1 and D1 strains of *T. foetus* were grown and maintained in 11 mL of TYM Diamond medium of pH 6.2. Every 24 h, the cells from the C1, D1, and G3 strains were passed by inoculating 1000 μL of cells (approximately 1 × 10^6^ cells) into a new 15 mL conical tube containing 10 mL of TYM Diamond medium. Then, the cells were incubated for 24 h at 37 °C. Inhibitory screens were carried out as previously described in several studies [8, 9, 14]. These assays were incubated at 37 °C for 24 h before being counted using a hemocytometer to count parasite motility as a measure of viability. Percentage inhibitory activities were calculated relative to the DMSO:water negative vehicle control at the same concentration as the test substances. There was very little if any toxicity associated with this DMSO:water solvent vehicle control. The positive control was metronidazole and was shown to be effective against all three trichomonad strains as has been reported [8].

#### Disc diffusion antibiotic sensitivity test of commensals and pathogens

*L. reuteri* (ATCC 23272), *L. acidophilus* (ATCC 43560), and *L. rhamnosus* (ATCC 53103) were grown in Lactobacilli MRS at 37 °C under anaerobic conditions using the BD GasPak EZ anaerobic container system. Strains grown aerobically at 37 °C were: *E. coli* K-12 MG 1655 (USDA) in Luria Broth, *S. enterica* pGFP (USDA) in Luria Broth, *L. monocytogenes* RM2194 (USDA) in Brain Heart Infusion, *B. cereus* (USDA) in Brain Heart Infusion, and *S. aureus* (ATCC 6538) in Tryptic Soy. Empty BDL-sensi-discs (6 mm) were incubated with either vehicle control (50% DMSO:50% water) or tomato powders dissolved in DMSO:water for 30 min at room temperature. Discs containing vehicle control, compounds, or various antibiotic discs (Oxoid antimicrobial sensitivity discs): levofloxacin (5 μg), gentamicin (10 μg), and gentamicin (120 μg), were placed onto the bacterial streaked agar plates and incubated overnight at 37 °C (18–24 h). Sensitivity to antibiotics or tomato powders was determined via measurement of zones of inhibition around each disc in millimeters (mm).

#### Fungal growth sensitivity assay

The antifungal activity of tomato peel extracts was examined in *Aspergillus fumigatus* AF293, a causative agent for invasive aspergillosis, and *C. albicans* ATCC 10231. In both *A. fumigatus* and *C. albicans* tests, 5 μL of 10% tomato peel powders w/v (dissolved in water: dimethyl sulfoxide) were spotted onto the lawn of fungi (in duplicate), which were grown on Potato Dextrose Agar (PDA) or Yeast Peptone Dextrose (YPD; Bacto yeast extract 1%, Bacto peptone 2%, glucose 2%) (Millipore Sigma, St. Louis, MO, USA) for *A. fumigatus* or *C. albicans*, respectively. Fungi were incubated at 35 °C and the formation of zones of inhibition in millimeters (mm) were monitored at 24 and 48 h.

#### Statistical analysis

All parasite screening trials were performed a minimum of three times on three separate days to a standard error of ≤0.10. Statistical significance was determined using the Student’s *t*-test to generate *p* values in the Prism 6 software (GraphPad, San Diego, CA, USA). *p* values < 0.05 were considered statistically significant.

## Results

### Inhibitory activity against human, bovine, and feline trichomonad parasites

Table 1 shows that the six tomato plant-derived powders, applied at final concentrations of 0.02% w/v, inhibited the growth of three pathogenic trichomonad strains to varying degrees. Percentage inhibitory activities were calculated relative to the DMSO:water negative vehicle control at the same concentration as the test substances. There was very little if any toxicity associated with this DMSO:water solvent vehicle control. The positive control was metronidazole which has shown to have an IC_50_ of 0.00000822% w/v (0.72 μM) (*T. vaginalis* G3), 0.00000559% w/v (0.49 μM) (T*. foetus* D1), and 0.00000628% w/v (0.55 μM) (*T. foetus* C1) [8]. Overall, each of the three parasites responded to the powders differently, although for certain powders, they shared a common response. For example, the feline strains C1 and bovine strain D1 responded nearly identically to tomato leaves and red tomato peels, but strikingly differently from each other for green tomato peels, while the inhibition of of the human D1 strain was more than 4× than either of the other 2 strains. Conversely, inhibition of strain D1 by the yellow tomato peel was about 1/7th the response of strains G3 and C1.

**Table 1:** Inhibition of parasite growth by tomato plant-derived powders^*a*^

Powders	***T. vaginalis*** G3 (human)	***T. foetus*** C1 (feline)	***T. foetus*** D1 (bovine)
Tomato leaves	70 ± 11	97.8 ± 2.6^*^	99.5 ± 2.4^*^
Tomato stems	38 ± 20	26.4 ± 8.6	44 ± 15
Green tomato peel	18 ± 1 1	20.1 ± 6.1^b^	89 ± 13^*^
Yellow tomato peel	43.3 ± 3.7	44.6 ± 4.5^b^	6.1 ± 1.9^*^
Red tomato peel	44.6 ± 7.5	19.3 ± 2.7^*^	19.4 ± 2.9^*^
Green tomato fruit	45.7 ± 9.6	36 ± 13	24.4 ± 3.3*

^*a*^The data represents the average % growth inhibition with standard deviations (SD) for each strain from three independent assays using the test powders at 0.02% w/v. Student’s *t-*test were performed for each powder to determine the statistical significance of the percent growth inhibition values for each of the three trichomonad strains to each other. *p* < 0.05 were considered statistically significant.* *p* < 0.05 for *T. foetus* C1 (feline) and *T. foetus* D1 (bovine) compared against *T. vaginalis* G3 (human); ^b^ *p* < 0.05 comparison between *T. foetus* D1 (bovine) and *T. foetus* C1 (feline)

Using the Student’s *t*-test for these percent inhibitory values for the tomato leaves extract for all trichomonad strains, we found a statistical difference between the *T. vaginalis* G3 (human) vs the *T. foetus* C1 (feline) and the *T. foetus* D1 (bovine) strain (Table 1, *p* < 0.05, denoted by ^*^). For the green tomato peel, it was found that there was a statistical difference between *T. vaginalis* G3 vs *T. foetus* D1(bovine) as denoted by the ^*^ and between *T. foetus* C1 (feline) vs *T. foetus* D1 (bovine) denoted by ^b^), (Table 1, *p* < 0.05). Similar to the green tomato peel, the yellow tomato peel powder was statistically significant with a *p* < 0.05 in Table 1 between *T. vaginalis* G3 vs *T. foetus* D1(bovine) and between *T. foetus* C1 (feline) vs *T. foetus* D1 (bovine). The red tomato peel was also shown to be statistically significant between the *T. vaginalis* G3 (human) vs the *T. foetus* C1 (feline) and the *T. foetus* D1 (bovine) strain (Table 1, *p* < 0.05, denoted by ^*^). Last, the green tomato powder itself was only found to be statistically significant between the *T. vaginalis* G3 (human) vs the *T. foetus* D1 (bovine) strain (Table 1, *p* < 0.05, denoted by ^*^).

Tomato leaves exhibited the highest activity across all strains, with nearly complete inhibition of strains C1 and D1 at values of 97.8 and 99.5%, respectively. This sample was then subjected to IC_50_ determination (concentration that inhibited 50% of the pathogens under the tesst conditions) for the three pathogens and determined to be approximately 0.02% (w/v) for strain G3, 0.01% (w/v) for strain C1, and 0.01% (w/v) for strain D1 when rounded to only one significant digit. Below we will discuss potential reasons for these differences in terms of the known composition of the test powders.

### Relationship between trichomonad growth inhibition and chemical composition of the tomato plant and fruit

**Fig. 1:**
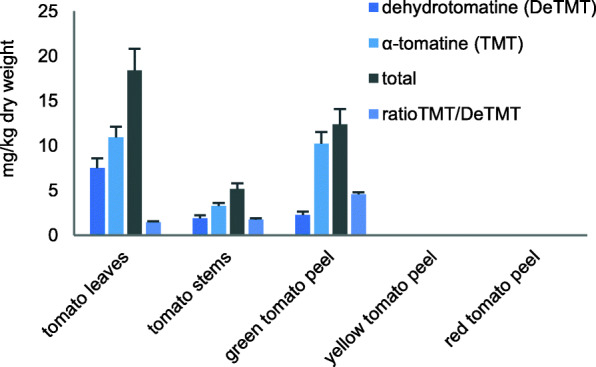
The α-tomatine and dehydrotomatine content and sums and ratios of both in five test powders with standard deviations. Adapted from Friedman, et al. [13]

**Fig. 2:**
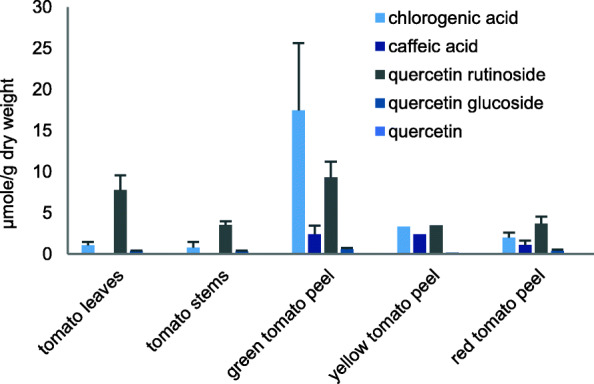
Phenolics measured in tomato powders in μmole/g dry weight. Adapted from Friedman, et al. [13]

We previously reported on the composition, as determined by high-performance liquid chromatography-mass spectrometry (HPLC-MS), of five of the test powders evaluated in the present study in terms of mg/g dry weight [13]. Figure 1 shows the trends in the concentrations of α-tomatine and dehydrotomatine in the powders. Tomato glycoalkaloids are known to be more prevalent in the vegetative part of the plant, and in the immature fruit. Glycoalkaloids in the fruit degrade during maturation. Thus, the peels of the yellow and red fruits contained no detectable glycoalkaloids, as expected. Figure 2 depicts the content of phenolic compounds in 5 powders analyzed in a previous study [13]. The phenolic acids were only prevalent in the fruit peels; the leaves and stems containing small amounts of chlorogenic acid, and no caffeic acid. Quercetin glycosides were present in all the samples, but only a trace amount of non-glycosylated quercetin was found in one sample, the yellow tomato peel. Quercetin glucosides were mostly rutinosides, as opposed to glucosides. The green tomato fruit peel contained a much larger amount of chlorogenic acid than any of the other samples, and along with leaves, a large amount of quercetin rutinoside.

### Growth inhibition of pathogenic bacteria

Table 2 shows the growth inhibition of four pathogenic bacteria and a common laboratory strain, *E. coli* K12 (used as a model for other pathogenic *E. coli*) by the antibiotics levofloxacin and gentamycin (used as positive controls) and five plant powders in a 10% w/v solution as an initial screen for anti-microbial activity. Tomato stems, red tomato peels, and green tomato fruit showed no activity against any of the bacteria. Tomato leaves and green tomato peel, both high in tomatine, inhibited *S. enterica* to a minor extent. Most of the other bacteria were not affected by the treatments, with the exceptions that tomato leaves alone inhibited *B. cereus*, and green tomato peels alone inhibited *E. coli* K12. Either the tomatine was not responsible for the activity against these 2 bacteria, or some other compound, perhaps the high chlorogenic acid content of green peels, was acting antagonistically or synergistically with the tomatine. The inhibition of *B. cereus* by the tomato leaf powder was on par with the 10 μg gentamycin treatment.

**Table 2:** Inhibition of bacteria by powders derived from a wild, drought-resistant tomato plant^*a*^

	Zones of inhibition (mm)							
	*E. coli* K12	*S. enterica*	*L. monocytogenes*	*S. aureus*	*B. cereus*	*L. acidophilus*	*L. rhamnosus* GG	*L. reuteri*
DMSO:water vehicle	0	0	0	0	0	0	0	0
Levofloxacin 5 μg	30	30	17	36	26	0	15	5
Gentamicin 10 μg	18	16	22	25	15	8	8	11
Gentamicin 120 μg	20	22	30	30	20	15	15	20
Tomato leaves	0	8*	0	0	13*	0	0	0
Tomato stem	0	0	0	0	0	0	0	0
Green tomato peel	8*	7*	0	0	0	0	0	0
Red tomato peel	0	0	0	0	0	0	0	0
Yellow tomato peel	0	0	0	0	0	0	0	0
Green tomato fruit	0	0	0	0	0	0	0	0

^*a*^A disc diffusion assay was performed with either vehicle, antibiotic controls, or tomato powders at 10% (w/v). * indicates sensitivity to the tomato powders

### The effect of test powders on non-pathogenic lactobacilli

Table 2 also shows that none of the seven test powders inhibited the growth of three beneficial lactobacilli, *L. acidophilus*, *L. rhamnosus*, and *L. reuteri*.

### Growth inhibition of pathogenic fungi

Table 3 shows the results of experiments to determine if the tomato powders might also inhibit the growth of two pathogenic fungi. None of the powders was effective against *Aspergillus fumigatus*, an environmental filamentous fungus that can cause life-threatening disease in immunocompromised individuals [15]. By contrast, Table 3 shows that tomato leaves, stems, and green tomato fruit inhibited the growth of the pathogenic fungus *C. albicans* to a significant extent (63–74%) relative to the positive control octyl gallate.

**Table 3:** Fungal pathogen growth sensitivity to tomato powders^*a*^

	Zones of inhibition (mm)	
Controls and test powders	*A. fumigatus* AF293	*C. albicans* ATCC 10231
DMSO:water (vehicle)	0	0
Octyl gallate (control) 0.117% w/v	10.5	17.8
Tomato leaves	0	13*
Tomato stem	0	13*
Green tomato peel	0	0
Red tomato peel	0	0
Yellow tomato peel	0	0
Green tomato fruit	0	11.5*

^a^The antifungal activity of tomato powders (10% w/v) was tested on *Aspergillus fumigatus* AF293 and *C. albicans* ATCC 10231. Zones of inhibition in mm were measured from the negative control vehicle (DMSO:water); positive control (0.117% w/v octyl gallate); and tomato powders. * indicates sensitivity to powders

## Discussion

Previous compositional studies have shown that dehydrotomatine is always present to a lesser degree than tomatine in tomato plants [16, 17]. While also true in this study (Fig. 1), the relative amount of dehydrotomatine to tomatine in plants powders (18–41% of total glycoalkaloids) was higher than previously reported values ranging from 5 to 18% [17]. It is possible this difference is due to improved analytical techniques since the 2004 paper, or alternatively to normal variation not previously discerned by the small sample pools analyzed. The ratio of tomatine to dehydrotomatine is potentially important because of reported differences in the biological effects of the two tomato glycoalkaloids [18]. We tested purified α-tomatine and dehydrotomatine, collected from a preparative HPLC column, and in limiting amounts, against the *Trichomonas vaginalis* and found α-tomatine had an approximate IC_50_ of 25 μM, while dehydrotomatine had no observable effect on cell growth [8].

Other than an apparent correlation between tomatine content of the plant powders and inhibition of strain D1, there does not appear to be any obvious patterns of correlation between composition and activity, possibly because plant components could act additively, antagonistically, or synergistically in binding to the cell receptor sites and because the analytical samples might not be identical to the biological samples, although both sets were harvested at the same time from the same plants. It is nevertheless of interest to note that tomato leaves, which have a high content of tomatine and dehydrotomatine, showed the greatest potency against all three trichomonad strains. This finding is consistent with our previous report [9], on the observed high potency of commercial tomatine against the three trichomonads. Inhibition of strain D1 appears to correlate with tomatine content of the powders, while inhibition of the other strains does not. It is also likely that the phenolic acids and the two quercetin glycosides contribute to the inhibitory activities, possibly accounting for the activity in yellow and red peels. Previously we reported that pure phenolic acids and flavonoids inactivated the trichomonads, although at a lower efficacy than observed for tomatine [10]. Also noteworthy is that the green tomato peel powder, also containing high tomatine, was less active against strains G3 and C1 than that of the leaf powder by a factor of more than 4. With the green peel being very high in chlorogenic acid, it is likely that the G3 and C1 strains are protected by the antioxidative effect of chlorogenic acid.

These observations suggest that because the biosynthesis of secondary metabolites change during the growth cycle of the plant [19], and the metabolites affect the potency of the powders unpredictably, it might not be possible to predict the anti-trichomonad effects of different plant materials based on component analysis. The most biologically active powders (especially those prepared from tomato leaves) against each strain merit further evaluation in in vivo domestic and farm animal, and human clinical studies to confirm the described in vitro results.

The results on the growth inhibition of the pathogenic bacteria in Table 2 suggest that some of the test powders have the potential to concurrently inhibit some pathogenic bacteria that might be associated with bacterial vaginosis, a disease sometimes misdiagnosed as trichomoniasis, as well as bacteria in contaminated human food and animal feed. However, these results were obtained using a high concentration (10% w/v) of the various powders as an initial screen for anti-microbial activity and thus are too high to be used clinically. Future studies will address this issue and refinement of the concentrations to be more clinically relevant and yet retain their anti-microbial effects is required.

The results shown in Table 2 on the lack of inhibition of the test substances on the three lactobacilli is a useful finding because, as mentioned elsewhere [20–22], lactobacilli are present in the natural microflora in the vagina and help maintain the normal acid pH, produce bactericides that help prevent infection, and reduce adherence of urogenital pathogens to host receptors. These considerations imply that the above-described anti-trichomonad effects of the test powders will not be adversely affected by the undesirable concurrent inhibition of useful lactobacilli.

The inhibition of *C. albicans* fungi shown in Table 3 is also a useful finding because this fungus is reported to cause the prevalent human vaginal infection commonly known as ‘yeast infection’. Although normally present in healthy humans, it grows out of control when the pH in the vagina increases. This can be brought on by various stresses or by oral antibiotics which can kill beneficial lactobacilli bacteria. *C. albicans* can also occasionally cause more serious systemic infections in severely ill or immuno-compromised individuals [23]. It is notable that green tomato peels were inactive against *Candida. albicans*, suggesting that the glycoalkaloids were either not responsible for the inhibition, or that another component in the green peels was interfering with activity. We do not know the reason for the differential susceptibilities of the two pathogenic fungi to growth inhibition. However, a similar phenomenon could be found with the antifungal agent fluconazole. The yeast pathogens *Candida* sp. and *Cryptococcus* sp. are susceptible to fluconazole whereas the filamentous fungal pathogen *Aspergillus fumigatus* whch was is not inactivated by any of the test substances is not, reviewed in [24]. This natural resistance might be linked to the naturally occurring T301I mutation in the cytochrome P450 enzyme gene encoding 14-α sterol demethylase A [25].

## Conclusions and research needs

This investigation has shown that powders prepared from different parts of a harvested tomato plant, especially high-tomatine content leaves, have in vitro anti-trichomonad, anti-bacterial, and anti-fungal properties, suggesting their possible value to ameliorate the severity of trichomoniasis, vaginosis, and vaginal yeast infections in infected humans and trichomonosis in infected farm and domestic animals. The results of the present study contribute to our knowledge about the biological properties of different parts of the tomato plant. Because the analytical data show that the tomato leaves and green tomatoes have the highest content of bioactive compounds compared to red tomatoes, future biomedical studies should consider evaluating health benefits of readily available and inexpensive tomato leaves from different tomato cultivars.

Our wild, drought-resistant tomato plant has the morphological characteristics of a “cherry” tomato plant but this identification is informal and future studies need to validate this preliminary determination via genomic analysis leading to the typing of the taxonomy of this plant. The results of the present study complement and extend related observations on the inhibition of the parasitic trichomonds by cherry (grape) tomato peels [14]. Moreover, the present and related studies also suggest the need for the following additional studies that might help broaden the scope and the dietary and medical value of the most active test powders: (a) Determine in clinical trials if readily available and inexpensive high-tomatine tomato plant powders such as tomato leaves might be effective against trichomoniasis in infected women [26, 27]; (b) Determine whether animal feed or human food supplemented with the tomato plant powders will result in functional diets that might help protect humans and animals against trichomoniasis and trichomonosis; (c) Define the possible efficacy of the powders against microbial and plant toxins [28]; (d) Determine if the reported inhibition of pathogenic viruses by tomatine [29], and by the aglycone tomatidine [30, 31], suggest that these two compounds and tomatine-containing tomato leaves and tomatine-containing wild potato cultivars [32], might also inhibit other pathogenic viruses such as coronaviruses and the human immunodeficiency virus (HIV); (e) Define the effectiveness of the tomato powders against metronidazole-resistant trichomonad strains; (f) Determine if tomato peel powders will be effective against cancer [33, 34], high plasma cholesterol and triglyceride levels [35, 36], obesity [37], and malaria [38]; (g) Determine if the chlorophyll in tomato leaves can contribute to the functional health benefits [39]; (h) The present successful proof-of-principle study suggests the need to determine the range of tomatine, phenolic, and flavonoid content in other varieties of tomato plants, including organically and conventionally grown standard and cherry tomatoes and with the objective of finding plants that biosynthesize high levels of the bioactive compounds [19]; (i) Using antisense RNA molecular biology methods [40], create high-tomatine red tomatoes by suppressing the genes that govern the biosynthesis of enzymes that degrade tomatine during maturation of high-tomatine green tomatoes to low-tomatine red ones; and (j) Evaluate antiparasitic activities of the readily available inexpensive potato leaves that contain the glycoalkaloids α-chaconine and α-solanine [41], which we reported to have anti-trichomonad properties [10].

We are challenged to help ameliorate adverse effects of pathogen contamination in food and infection in animals and humans using extracts containing bioactive compounds present in different parts of the tomato plant. In vivo studies should include an assessment of the ratio of effective to toxic doses.

## Data Availability

The data used and/or analyzed during the current study are available from the corresponding author on reasonable request.

## Abbreviations

HFD: high-fat diet
ROS: reactive oxygen species
MMP: mitochondrial membrane potential
MDI: MIX-DEX-Insulin differentiation medium
AMPK: adenosine 5′-monophosphate-activated protein kinase
PPARγ: peroxisome proliferator activated receptor-γ
C/EBPα: CCAAT enhancer binding protein α
RNA: ribonucleic acid
DMSO: dimethyl sulfoxide
